# Agreement between 24-hour urine and 24-hour food recall in measuring salt intake in primary school children in Australia

**DOI:** 10.1186/s12937-022-00823-8

**Published:** 2022-11-15

**Authors:** Joseph Alvin Santos, Kristy A. Bolton, Emalie Rosewarne, Kathy Trieu, Gian Luca Di Tanna, Mark Woodward, Jacqui Webster, Carley Grimes

**Affiliations:** 1 The George Institute for Global Health, University of New South Wales, Newtown, NSW 2042 Australia; 2 Institute for Physical Activity and Nutrition, School of Exercise and Nutrition Sciences, Deakin University, Geelong, VIC 3220 Australia; 3 Department of Innovative Technologies, University of Applied Sciences and Arts of Southern Switzerland, Lugano, Switzerland; 4 The George Institute for Global Health, School of Public Health, Imperial College London, London, UK

**Keywords:** Salt intake, 24-hour urine, 24-hour food recall

## Abstract

**Background:**

Monitoring salt consumption in children is essential for informing and implementing public health interventions to reduce children’s salt intake. However, collection of 24-hour urines, considered as the most reliable approach, can be especially challenging to school children. This study aimed to assess the agreement between 24-hour urine (24hrU) and 24-hour food recall (24hrFR) in: (1) estimating salt intake in children; (2) classifying salt intakes above the recommended upper level set for children, and; (3) estimating change in mean salt intake over time.

**Methods:**

This study utilised data from two cross-sectional surveys of school children aged 8 to 12 years living in the state of Victoria, Australia. A single 24hrU and 24hrFR were collected from each participant. Suspected inaccurate urine collections and implausible energy intakes were excluded based on pre-defined criteria. The agreement between the two methods was assessed using Bland-Altman methodology, the intraclass correlation coefficient (ICC), and the kappa statistic. The difference between the measured change in salt intake over time using 24hrU and 24hrFR was derived using mixed effects linear regression analysis.

**Results:**

A total of 588 participants provided a 24hrU and 24hrFR. Overall, there was no meaningful difference in mean estimated salt intake between the two methods (− 0.2 g/day, 95% CI − 0.5 to 0.1). The Bland-Altman plot showed wide 95% limits of agreement (− 7.2 to 6.8). The ICC between the two methods was 0.13 (95% CI 0.05 to 0.21). There was poor interrater reliability in terms of classifying salt intake above the recommended upper level for children, with an observed agreement of 63% and kappa statistic of 0.11. The change in mean salt intake over time was 0.2 g/day (− 0.4 to 0.7) based on 24hrU, and 0.5 g/day (− 0.0 to 1.1) based on 24hrFR, with a difference-in-differences of 0.4 g/day (− 0.3 to 1.1).

**Conclusions:**

24hrFR appears to provide a reasonable estimate of mean salt intake as measured by 24hrU in Australian school children. However, similar to previous observations in adults, and of studies exploring other alternative methods for estimating salt intake, 24hrFR is a poor predictor of individual-level salt intake in children.

**Supplementary Information:**

The online version contains supplementary material available at 10.1186/s12937-022-00823-8.

## Background

It is recommended that children consume less salt than adults [1], and in many countries, children consume more than the recommended limit of salt per day [2–6]. Evidence from systematic reviews and meta-analyses shows that high salt intake in children leads to high blood pressure (BP), which tracks across the life course [7–9], and that elevated childhood BP is associated with the development of hypertension and related cardiovascular outcomes in adulthood [10]. It is thus essential to quantify and monitor salt consumption in children, as well as identify children’s dietary sources of salt, to inform and implement public health interventions to reduce children’s salt intake.

Twenty-four urine collection (24hrU) is preferred over other assessment methods for measuring individual- or population-level salt intake [11]. It involves collection of all urine over a full 24-hour period–a process considered to be burdensome and prone to inaccurate collections [12]. In addition, the method suffers from the high day-to-day variability in sodium excretion so multiple measurements is recommended [13, 14], although in practice this is costly and difficult to attain. Among children, collection of 24-hour urine samples can be especially challenging, particularly to those who attend schools who might find it inconvenient to carry containers and collect urine samples outside the home [15]. For these reasons, alternative approaches for measuring salt intake have been explored, including dietary-based assessment methods (such as 24-hour food recall, food frequency questionnaire or food diaries) and other urine-based methods (such as spot urine or overnight urine samples) [12].

Recent systematic reviews and meta-analyses comparing salt intake estimates from the above mentioned alternative approaches with 24hrU revealed that these approaches are inadequate for assessing individual-level salt intake [16–19]. In terms of population-level salt intake, spot urine samples provided a reasonable estimate of mean salt intake compared to a single 24hrU [19], while 24-hour food recall (24hrFR) underestimated mean salt intake, with smaller difference from 24hrU observed in studies conducted in high-income countries [17]. However, it must be noted that while some studies have suggested that spot urines can produce comparable mean salt intake estimates with 24hrU at a single time point, there remains uncertainty about its capacity to measure changes in salt intake over time [20]. Yet another issue is that previous studies on accurate assessment of mean salt intake (in particular those that pooled data from multiple sources) focused only on adults [16–20], and little is known how these findings apply to children. Clearly, there remains several questions regarding the extent to which the alternative approaches are capable of producing reliable estimates and monitoring population changes in salt intake. Therefore, this study aimed to assess the agreement between 24hrU and 24hrFR in: (1) measuring salt intake in children; (2) classifying salt intakes above the recommended upper level set for children, and; (3) estimating change in mean salt intake over time.

## Methods

This study utilised data from two cross-sectional surveys of primary schoolchildren aged 8 to 12 years living in the state of Victoria, Australia. The surveys were conducted before (2010–2013) and after (2018–2019) the implementation of the Victorian Salt Reduction Partnership’s state-wide intervention which aimed to reduce population salt intake among children and adults [21]. The study protocol [22] and evaluation of the interventions [23–25] have been published elsewhere.

### Recruitment of participants and collection of 24HUNa and 24HFR

The strategy for selecting schools and schoolchildren have been described previously [22]. Briefly, in each survey, a convenience sample of government and non-government primary schools was selected, and children were invited to participate in the study. At follow-up, schools and children were recruited to match the sample included at baseline according to the type (i.e. government or non-government) and socio-economic disadvantage level of the school. Given that children’s diet, including salt intake, vary by socioeconomic background [26], these two variables were deemed important as indicators of children’s socioeconomic profile.

In both the baseline and follow-up surveys, a single 24hrU and 24hrFR were collected from children who agreed to participate. Written instructions (with pictures) on how to properly collect the sample were provided to children and their parents. On the day of collection, children discarded their first urine void and collected all subsequent urine in the following 24-hour period. Children were asked to report any missed voids or spillage. Urine samples were sent to the laboratory for volume, sodium, potassium, and creatinine concentration analysis [22]. For 24hrFR, children reported their intakes and the multiple-pass method (i.e. face-to-face 3-pass method at baseline [22] and web-based 5-pass method at follow-up [27]) was employed. Recognizing that 24hrFR is prone to bias due to memory lapses or inaccurate measurement of portion sizes even more so among children [12, 28], the children were assisted by an interviewer to recall their food intakes, and the interviewer recorded the food items consumed (on paper at baseline and directly into the ASA-24 online software at follow-up). Nutrient intakes were calculated using the Australian Food and Nutrients Database 2011–2013 [29]. Children were given the option to collect the urine on either a school or non-school day while the food recalls were collected within the school, hence, the days in which the 24hrU and 24hrFR were completed varied (e.g. some on the same day, others on different days) dependent on participant’s preferences for urine data collection and scheduling of 24hrFR at schools.

### Data processing

Only children with both 24hrU and 24hrFR were included in the analysis. Furthermore, only 24hrFRs from children aged 8 years and above were used in the analyses as it is considered from this age children have the capacity to self-sufficiently recall their food intakes during the previous day [22, 30]. For 24hrU, urine volume was standardised to a 24-hour period if the collection duration was not exactly 24 hours but within 20 to 28 hours [22]. In the main analysis, suspected inaccurate urine collections were excluded based on the following criteria: (1) volume < 300 mL/day; (2) creatinine excretion < 0.1 mmol/kg/day; (3) collection time < 20 or > 28 hours, and; (4) participant missed > 1 collection [22]. Twelve other criteria for assessing completeness of 24hrU were used in the sensitivity analysis to assess the robustness of the main findings (Additional file 1). For 24hrFR, participants who did not complete the recall (e.g. could not remember an entire meal or indicate quantities consumed) or those with implausible energy intakes were excluded. Implausible energy intakes were assessed using the *energy intake to estimated basal metabolic rate* (EI:estBMR) ratio of < 0.87 for boys < 0.84 for girls [22]. Outliers for energy and sodium intake (> 4 SDs from the mean) were excluded. Salt intake estimates from both 24hrU and 24hrFR were reported in g/day.

### Data analysis

For assessing the agreement between the two methods, the baseline and follow-up samples were combined. The agreement was assessed using Bland-Altman methodology [31] and two-way mixed effects intraclass correlation coefficient (ICC, which measures the correlation between 24hrU and 24hrFR measurements on the same participant) [32]. Regression-based 95% limits of agreement were added to the Bland-Altman plot to determine whether the agreement between the two methods varied according to the level of salt intake [33]. Subgroup analyses were conducted to determine whether the agreement between the two methods would vary according to: (1) the type of day the 24hrU and 24hrFR were collected (i.e. school or non-school day), and; (2) the number of days between the collection of 24hrU and 24hrFR.

The kappa statistic [34] was used to determine the interrater reliability between 24hrU and 24hrFR in classifying salt intake above 5 g/day (for children aged 9 to 12 years) and 3.5 g/day (for children aged 8 years) [35]. For comparing the estimates of change in mean salt intake between the two methods, the baseline and follow-up data were used separately. The difference between the measured change in salt intake over time using 24hrU and 24hrFR (i.e. difference-in-differences) was assessed using mixed effects linear regression analysis. Statistical analyses were carried out using Stata IC V15.1 for Windows (StataCorp LLC, College Station, TX).

## Results

**Fig. 1:**
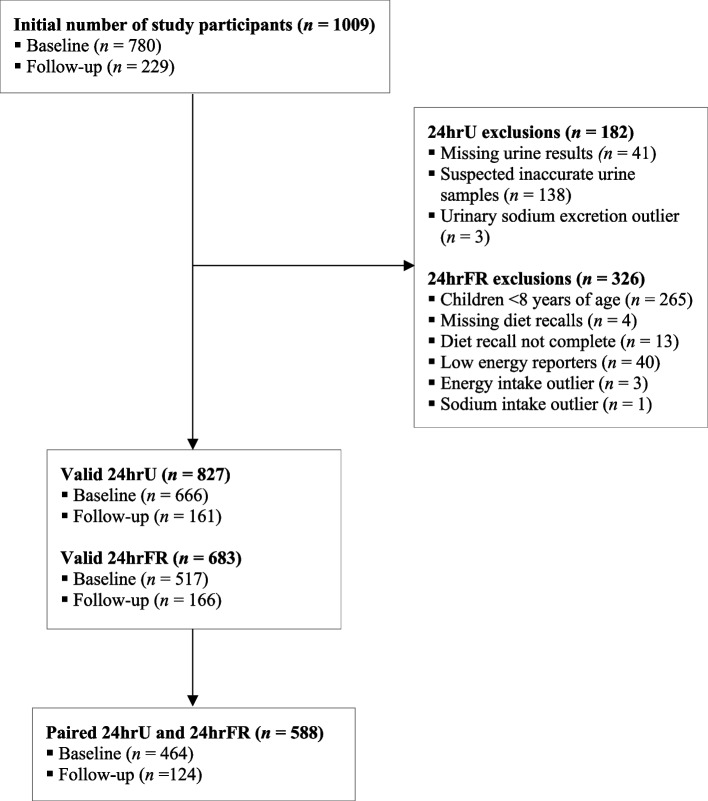
Flowchart of participants included in the analyses

A total of 1009 children (780 at baseline and 229 at follow-up) participated in the surveys. Of these, 827 and 683 children provided complete 24hrU and 24hrFR, respectively. The reasons for exclusion of 24hrU and 24hrFR are shown in Fig. 1. Ultimately, 588 paired 24hrU and 24hrFR data were included.

The characteristics of participants are shown in Table 1. The mean age of the sample was 10 years, with about an equal number of boys and girls. Approximately 75% of the children had healthy weight. There were no differences in terms of age and anthropometric measures by sex. However, boys had higher 24-hour urine volume (by 100 mL) and urine creatinine excretion (by 0.6 mmol/day) than girls. More than half and about a quarter of the children completed the 24hrU and 24hrFR on a non-school day, respectively.

**Table 1:** Characteristics of participants

Characteristic	Total (*n* = 588)	Girls (*n* = 273)	Boys (*n* = 315)
Age, years (mean, SD)	10.2 (1.2)	10.1 (1.2)	10.3 (1.3)
BMI z-score^a^ (mean, SD)	0.2 (1.0)	0.2 (1.0)	0.2 (0.9)
Weight category^b^ (n, %)			
Underweight	39 (6.6)	22 (8.1)	17 (5.4)
Healthy weight	443 (75.3)	194 (71.1)	249 (79.1)
Overweight/obese	106 (18.0)	57 (20.9)	49 (15.6)
Hip circumference, cm (mean, SD)	75.5 (8.4)	75.5 (8.4)	75.5 (8.3)
Waist circumference, cm (mean, SD)	66.2 (9.1)	66.1 (9.4)	66.3 (8.9)
Waist-to-Hip ratio (mean, SD)	0.5 (0.1)	0.5 (0.1)	0.5 (0.1)
Urine volume, mL (mean, SD)	900.4 (432.6)	846.8 (394.2)	946.9 (458.8)
Urine creatinine, mmol/day (mean, SD)	6.0 (1.9)	5.7 (1.8)	6.3 (1.9)
Participants who completed 24hrU on a non-school day (n, %)	312 (53.1)	149 (54.6)	163 (51.8)
Participants who completed 24hrFR on a non-school day (n, %)	137 (23.3)	65 (23.8)	72 (22.9)

^a^ BMI z-scores were calculated using the 2000 US Centers for Disease Control and Prevention growth charts [36]

^b^ Participants were grouped into weight categories using the International Obesity Taskforce BMI reference cut-offs for children [37]

### Mean salt intake estimated from 24hrU and 24hrFR

The mean salt intake measured using 24hrU was 6.4 g/day (95% CI 6.2 to 6.6), while the corresponding mean salt intake estimated using 24hrFR was 6.2 g/day (95% CI 6.0 to 6.4). Overall, there was no difference in mean salt intake between the two methods (− 0.2 g/day, 95% CI − 0.5 to 0.1) (Table 2). Subgroup analyses showed that 24hrFR underestimated 24hrU by about − 0.4 g (95% CI − 0.8 to − 0.0) when it was not collected on the same type of day (school or non-school day) as the 24hrU, and by about -1 g (95% CI − 1.5 to − 0.5) when it was collected within 4–7 days of the urine collection. On the other hand, 24hrFR overestimated 24hrU by 0.4 g (95% CI 0.1 to 0.8) when it was collected within 1–3 days of the urine collection.

**Table 2:** Difference in mean salt intake between 24hrU and 24hrFR, overall and by timing of collection

	24hrU		24hrFR		Difference	
	Mean	95% CI	Mean	95% CI	Mean	95% CI
**Overall (*n*** **= 588)**	**6.4**	6.2 to 6.6	**6.2**	6.0 to 6.4	**− 0.2**	0.5 to 0.1
**By type of day (school or non-school day)**						
Collected on the same type of day (*n* = 279)	**6.2**	5.9 to 6.5	**6.2**	5.9 to 6.6	**0.0**	−0.4 to 0.4
Not collected on the same type of day (*n* = 309)	**6.6**	6.3 to 6.9	**6.1**	5.8 to 6.4	**−0.4**	−0.8 to − 0.0
**By number of days between collection**						
Same day (*n* = 31)	**7.3**	6.5 to 8.2	**6.7**	5.4 to 8.1	**−0.6**	−2.1 to 1.0
Within 1–3 days (*n* = 306)	**5.8**	5.5 to 6.1	**6.2**	5.9 to 6.5	**0.4**	0.1 to 0.8
Within 4–7 days (*n* = 201)	**7.1**	6.7 to 7.5	**6.1**	5.7 to 6.5	**−1.0**	−1.5 to −0.5
>7 days (*n* = 51)	**6.9**	6.0 to 7.8	**5.9**	5.3 to 6.6	**−1.0**	−2.1 to 0.1

**Fig. 2:**
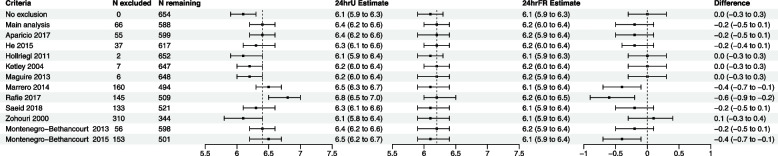
Salt intake estimates measured using 24hrU and 24hrFR applying various criteria for assessing 24hrU completeness

The sensitivity analyses showed that the use of other criteria for assessing completeness of 24hrU resulted in varying sample sizes for analysis, but most produced comparable results to the main analysis apart from three criteria (Fig. 2).

### Agreement between 24hrU and 24hrFR

**Fig. 3:**
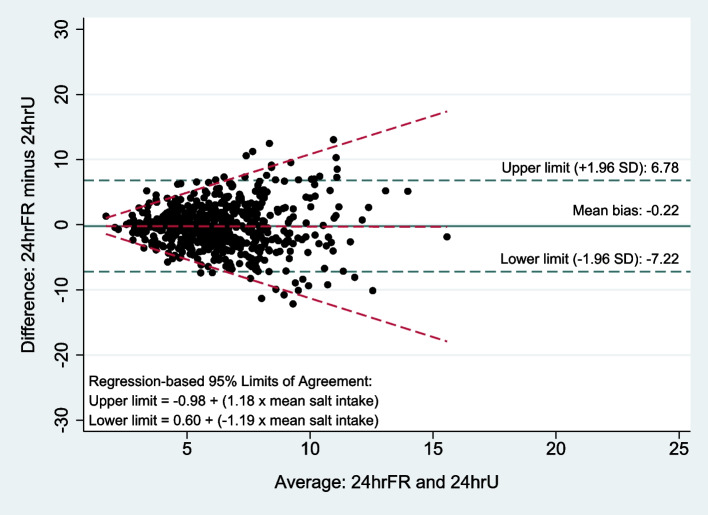
Bland-Altman plot of salt intake measured using 24hrU and 24hrFR

Figure 3 illustrates the Bland-Altman plot of salt intake measured using 24hrU and 24hrFR. The plot shows wide limits of agreement (LoA) between the two methods (mean bias − 0.2, 95% LoA − 7.2 to 6.8). The regression-based lines also demonstrate widening of LoA with increasing levels of salt intake. On the other hand, the ICC between the two methods was 0.13 (95% CI 0.05 to 0.21), suggesting poor reliability between the two methods in estimating individual-level salt intake. In terms of classifying salt intake as above or below the recommended limits for children, 24hrU showed that 68% had salt intakes above the limits while the corresponding proportion for 24hrFR was 67%. However, the observed agreement was 63% with a kappa statistic of 0.113, suggesting poor interrater reliability between the two methods in classifying salt intakes at the individual level. The subgroup analyses by type of day and by number of days between collection of 24hrU and 24hrFR showed poor interrater reliability across all subgroups (ICC range of − 0.08 to 0.21 and kappa statistic range of − 0.228 to 0.161). The lowest ICC and kappa statistic were observed in the subgroup where the number of days between collection of 24hrU and 24hrFR was greater than seven days (Additional file 2).

### Change in salt intake over time

The change in mean salt intake over time was 0.2 g/day (95% CI − 0.4 to 0.7) based on 24hrU, and 0.5 g/day (95% CI − 0.0 to 1.1) based on 24hrFR, with a difference-in-differences between the two methods of 0.4 g/day (95% CI − 0.3 to 1.1).

## Discussion

These analyses provided an in-depth assessment of the agreement between 24hrU and 24hrFR in measuring salt intake in primary school children living in Victoria, Australia. The results indicated that 24hrFR produced a reasonable estimate of mean salt intake compared to 24hrU, but performed poorly in terms of estimating individual-level salt intake. This is in line with the emerging consensus among adults that alternative methods for measuring salt intake are poor predictors of individual-level intake, but could provide adequate estimates of intake in groups or populations [16–19, 38]. The magnitude of the difference in mean salt intake between 24hrU and 24hrFR in these analyses (about 0.2 g/day) was notably lower compared to the difference previously observed in older populations (about 0.9 g/day when including studies that reported multiple-pass approach [17]). Subgroup analyses also showed that the timing of collection was an important factor, such that 24hrU and 24hrFR collected on the same type of day (school or non-school day) or on the same day had better agreement compared to samples collected on different days. These findings suggest the potential for 24hrFR to usefully estimate population-level salt intake in children, although more studies where both 24hrU and 24hrFR are collected, and ideally on the same day, are needed to strengthen these findings.

The smaller difference in mean salt intake between 24hrU and 24hrFR observed in this study compared to other similar studies among adults is noteworthy. This could be due to several reasons. Firstly, it is speculated that school-aged children consume less discretionary salt than adults (i.e. salt added at the table or during cooking, which is difficult to measure and often underestimated by 24hrFR [12]). Secondly, the multiple-pass method was used to collect dietary data at both baseline and follow-up, and a previous meta-analysis showed that studies that used multiple-pass methods demonstrated closer mean salt intake estimates between 24hrU and 24hrFR [17]. Thirdly, the same meta-analysis showed that there was better agreement between 24hrU and 24hrFR in studies carried out in high-income countries, which might be related to lower discretionary salt consumption in these countries than others.

The tendency of 24hrFR to underestimate mean salt intake as shown in adult studies [17] seemed to also apply to children. The measured mean salt intakes from 24hrFR in the main and sensitivity analyses were lower by 0.2 to 0.6 g/day than the salt intake estimates from 24hrU, although most were not *statistically significant*. Out of the 13 criteria used to assess completeness of urine samples, three showed that 24hrFR *significantly* underestimated 24hrU. It must be noted, however, that these three criteria were stricter compared to others (for example, using a stricter cut-off for urine volume and excluding participants with any missed voids [3, 39, 40]), leading to more urine samples being excluded from the analyses and possibly changing the sample composition. These observations are important in two ways: first, the tendency of 24hrFR to underestimate mean salt intake means that statistical adjustments might be possible to correct for the degree of underestimation of mean salt intake in children, and; second, given that there is no standard for assessing completeness of 24hrU, exploring different criteria is a useful exercise to confirm the robustness of the main analysis and ensure that applying different criteria leads to the same conclusions. It must also be noted that across the sensitivity analyses conducted, mean salt intake in children (i.e. 6.4 g/day from 24hrU) is higher than the recommended daily limit of 3.5 g/day and 5 g/day for 4–8 and 9–13 year old children, respectively, and is about the same as adult’s mean salt intake measured in the same study. This clearly supports the need for continued efforts to reduce salt intake in children.

The Bland-Altman plot and analysis, ICC, and kappa statistic consistently demonstrated that 24hrFR is a poor predictor of individual-level salt intake. The Bland-Altman analysis showed wide LoA between 24hrU and 24hrFR and also widening of LoA with increasing levels of salt intake. The degree of disagreement between the two methods was higher in children with higher salt intake, which means that children with higher salt intake under or overestimated their salt intake in the 24hrFR to a larger extent. Interestingly, there was no evidence of proportional bias (i.e. 24hrFR overestimate salt intake when actual consumption is lower, and underestimate salt intake when actual consumption is higher [33]), which is commonly observed in studies in adults comparing estimates from spot urine samples and 24hrU [19, 38, 41]. The absence of proportional bias suggests that 24hrFR might be able to perform better compared to spot urines in terms of measuring changes in mean salt intake over time [20, 42], although additional analyses are needed to quantify this. The ICC and kappa statistic showed poor interrater reliability between the two methods overall and across all subgroups, although 24hrU and 24hrFR collected more than 7 days apart showed the lowest interrater reliability. At the population-level, the proportion of children identified to have exceeded the recommended upper limit of salt intake was about the same for the two methods (i.e. about 70% exceeded the limit); however, at the individual level, the *correctly classified* intake (i.e. exact match between the two methods) was only 63%. This degree of misclassification at the individual-level raises some questions about the validity of using 24hrFR in carrying out analyses looking at the association of salt intake with disease risk.

The comparison of change in mean salt intake over time showed absence of a difference between the two methods. It must be noted that there was greater imprecision in this difference-in-differences analysis, given the smaller sample sizes at the individual time points. In addition, while the collection of 24hrFR at baseline and follow-up was both interviewer-assisted, the methods slightly differed such that a 3-pass method recorded on paper was used at baseline, while a 5-pass method recorded directly into an online software was used at follow-up. Surprisingly, the comparison of baseline average salt intakes showed that 24hrFR underestimated 24hrU by about 0.3 g/day, while the comparison of follow-up average salt intakes showed a smaller difference of about 0.1 g/day, despite the fact that there was a much higher sample size at baseline than at follow-up (464 and 124, respectively), with the follow-up data collection being impacted by the Covid-19 pandemic. This suggests the complexity of using 24hrFR in estimating change in mean salt intake in children, and highlights the need for reliability studies and further research prior to its application in a population.

The application of a number of analytical approaches to assess the agreement between 24hrU and 24hrFR is a strength of this study. In addition, the use of various criteria for assessing completeness of 24hrU, and the exclusion of low energy reporters based on 24hrFR, established the robustness of the main findings. The use of both baseline and follow-up data (and combining them) maximised the sample size available for the main analysis, although there was an uneven and smaller sample size available for the subgroup analyses and the analysis of change in salt intake over time. The limitations of the study include the use of a single 24hrU as the standard, which does not take into account the high day-to-day variability in sodium excretion [13, 14]. Another limitation is that not all participants completed the collection of 24hrU and 24hFR on the same day, which could have influenced the individual-level agreement observed between the two methods. The inclusion of children aged 8 to 12 years is also deemed as a limitation, as this means that the conclusions of this study might not be applicable to all primary school-aged children. Lastly, the use of different 24hrFR collection at baseline and follow-up could have affected the observed agreement between 24hrU and 24hrFR.

## Conclusion

In conclusion, these analyses suggest that 24hrFR may provide a reasonable estimate of mean salt intake compared to 24hrU in primary school children in Australia. However, similar to the observations in adults and of studies exploring other alternative methods for measuring salt intake, 24hrFR is a poor predictor of individual-level salt intake. The capacity of 24hrFR to estimate change in mean salt intake over time needs further investigation. Parallel collection of 24hrU and 24hrFR is needed to allow for a more in-depth assessment of the capacity of 24hrFR in estimating salt intake in children. This will be important for implementing population-based interventions to reduce salt intake and for adequately monitoring salt intake in children.

## Supplementary Information

**Additional file 1.** Criteria for assessing completeness of 24-hour urine samples.

**Additional file 2.** Intraclass correlation coefficients and kappa statistic by subgroups.

## Data Availability

The data that support the findings of this study are available from Dr. Carley Grimes at Deakin University but restrictions apply to the availability of these data, which were used under license for the current study, and so are not publicly available. Data are however available from the authors upon reasonable request and with permission of the Deakin University Human Research Ethics Committee.

## Abbreviations

BP: Blood pressure
24hrU: 24-hour urine collection
24hrFR: 24-hour food recall
EI:estBMR: Energy intake to estimated basal metabolic rate
ICC: Intraclass correlation coefficient
LoA: Limits of agreement
