# Supplementary material for: Agreement between 24-hour urine and 24-hour food recall in measuring salt intake in primary school children in Australia
Source: Nutr J. 2022 Nov 15;21:68. doi: 10.1186/s12937-022-00823-8 (PMC9664040; doi:10.1186/s12937-022-00823-8)
Supplement: Supplementary file 1 — Additional file 1. Criteria for assessing completeness of 24-hour urine samples. [file 12937_2022_823_MOESM1_ESM.docx]

**Table S1** Criteria for assessing completeness of 24-hour urine samples

| **Criteria** | **N excluded** | **N remaining** |
| --- | --- | --- |
| **1. Grimes 2016 [1]***   - Missed >1 collection or - Collection time <20h or >28h or - Urine volume <300ml/d or - Creatinine excretion <0.1mmol/kg/d | 66 | 588 |
| **2. No exclusion** | 0 | 654 |
| **3. Aparicio 2017 [2]**   - Creatinine excretion <0.1mmol/kg/d | 55 | 599 |
| **4. He 2015 [3]**   - Urine volume <300ml/d or - Creatinine excretion <5^th^ centile (<3.2mmol/d for boys and <2.8mmol/d for girls) | 37 | 617 |
| **5. Hollriegl 2011 [4]**   - Creatinine concentration <0.2 or >3.0g/L | 2 | 652 |
| **6. Ketley 2004 [5]**   - Urine volume <9ml/h or >420ml/h | 7 | 647 |
| **7. Maguire 2013 [6]**   - Urine volume <5ml/h for <6yo or <9ml/h for ≥6yo and - Creatinine excretion <11.3 or >21.1mg/kg/d | 6 | 648 |
| **8. Marrero 2014 [7]**   - Missed at least 1 collection or - Collection time <20h or >28h or - Urine volume <0.5ml/kg/h for 5-6 and 8-9yo or <500ml/d for 13-17yo or - Creatinine excretion <0.1mmol/kg/d | 160 | 494 |
| **9. Rafie 2017 [8]**   - Urine volume <500ml/d or - Creatinine excretion <0.1mmol/kg/d | 145 | 509 |
| **10. Saeid 2018 [9]**   - Missed more than a few drops of urine or - Collection time <20h or >28h or - Urine volume <300ml/d or - Creatinine concentration <280 or >2590mg/l | 133 | 521 |
| **11. Zohouri 2000 [10]**   - Creatinine excretion <14 or >20mg/kg/d | 310 | 344 |
| **12. Montenegro-Bethancourt 2013 [11]**   - Collection time <20h - Creatinine excretion <0.1mmol/kg/d | 56 | 598 |
| **13.** **Montenegro-Bethancourt 2015 [12]**   - Any missed collection - Creatinine excretion <0.1mmol/kg/d | 153 | 501 |

* main analysis

**Supplementary references:**

1. Grimes CA, Riddell LJ, Campbell KJ, He FJ, Nowson CA. 24-h urinary sodium excretion is associated with obesity in a cross-sectional sample of Australian schoolchildren. British Journal of Nutrition. 2016;115(6):1071-9.

2. Aparicio A, Rodríguez-Rodríguez E, Cuadrado-Soto E, Navia B, López-Sobaler AM, Ortega RM. Estimation of salt intake assessed by urinary excretion of sodium over 24 h in Spanish subjects aged 7-11 years. European journal of nutrition. 2017;56(1):171-8.

3. He FJ, Wu Y, Feng X-X, Ma J, Ma Y, Wang H, et al. School based education programme to reduce salt intake in children and their families (School-EduSalt): cluster randomised controlled trial. BMJ : British Medical Journal. 2015;350:h770.

4. Höllriegl V, Arogunjo AM, Giussani A, Michalke B, Oeh U. Daily urinary excretion of uranium in members of the public of Southwest Nigeria. The Science of the total environment. 2011;412-413:344-50.

5. Ketley CE, Cochran JA, Holbrook WP, Sanches L, van Loveren C, Oila AM, et al. Urinary fluoride excretion by preschool children in six European countries. Community dentistry and oral epidemiology. 2004;32 Suppl 1:62-8.

6. Maguire A, Walls R, Steen N, Teasdale L, Landes D, Omid N, et al. Urinary fluoride excretion in 6- to 7-year-olds ingesting milk containing 0.5 or 0.9 mg fluoride. Caries research. 2013;47(4):291-8.

7. Marrero NM, He FJ, Whincup P, Macgregor GA. Salt intake of children and adolescents in South London: consumption levels and dietary sources. Hypertension (Dallas, Tex : 1979). 2014;63(5):1026-32.

8. Rafie N, Mohammadifard N, Khosravi A, Feizi A, Safavi SM. Relationship of sodium intake with obesity among Iranian children and adolescents. ARYA Atheroscler. 2017;13(1):1-6.

9. Saeid N, Elmzibri M, Hamrani A, Latifa Q, Belghiti H, El Berri H, et al. Assessment of Sodium and Potassium Intakes in Children Aged 6 to 18 Years by 24 h Urinary Excretion in City of Rabat, Morocco. Journal of nutrition and metabolism. 2018;2018:8687192.

10. Zohouri FV, Rugg-Gunn AJ. Total fluoride intake and urinary excretion in 4-year-old Iranian children residing in low-fluoride areas. The British journal of nutrition. 2000;83(1):15-25.

11. Montenegro-Bethancourt G, Johner SA, Remer T. Contribution of fruit and vegetable intake to hydration status in schoolchildren. The American journal of clinical nutrition. 2013;98(4):1103-12.

12. Montenegro-Bethancourt G, Johner SA, Stehle P, Neubert A, Remer T. Iodine status assessment in children: spot urine iodine concentration reasonably reflects true twenty-four-hour iodine excretion only when scaled to creatinine. Thyroid : official journal of the American Thyroid Association. 2015;25(6):688-97.
