# Supplementary material for: Agreement between 24-hour urine and 24-hour food recall in measuring salt intake in primary school children in Australia
Source: Nutr J. 2022 Nov 15;21:68. doi: 10.1186/s12937-022-00823-8 (PMC9664040; doi:10.1186/s12937-022-00823-8)
Supplement: Supplementary file 2 — Additional file 2. Intraclass correlation coefficients and kappa statistic by subgroups. [file 12937_2022_823_MOESM2_ESM.docx]

**Table S2** Intraclass correlation coefficients and kappa statistic by subgroups

|  | **ICC (95% CI)** | **Observed agreement** | **Kappa statistic** |
| --- | --- | --- | --- |
| **Overall (*n* = 588)** | 0.13 (0.05 to 0.21) | 63% | 0.113 |
| **By type of day (school or non-school day)** |  |  |  |
| Collected on the same type of day (*n* = 279) | 0.15 (0.03 to 0.26) | 63% | 0.143 |
| Not collected on the same type of day (*n* = 309) | 0.11 (0.00 to 0.22) | 62% | 0.083 |
| **By number of days between collection** |  |  |  |
| Same day (*n* = 31) | 0.05 (-0.31 to 0.39) | 70% | 0.151 |
| Within 1-3 days (*n* = 306) | 0.13 (0.02 to 0.24) | 62% | 0.161 |
| Within 4-7 days (*n* = 201) | 0.21 (0.08 to 0.34) | 65% | 0.104 |
| >7 days (*n* = 51) | -0.08 (-0.35 to 0.20) | 49% | -0.228 |
